# A bifunctional cerium phosphate catalyst for chemoselective acetalization†
†Electronic supplementary information (ESI) available. See DOI: 10.1039/c6sc05642c



**DOI:** 10.1039/c6sc05642c

**Published:** 2017-02-07

**Authors:** Shunsuke Kanai, Ippei Nagahara, Yusuke Kita, Keigo Kamata, Michikazu Hara

**Affiliations:** a Laboratory for Materials and Structures , Institute of Innovative Research , Tokyo Institute of Technology , Nagatsuta-cho 4259, Midori-ku , Yokohama 226-8503 , Japan . Email: hara.m.ae@m.titech.ac.jp; b Advanced Low Carbon Technology Research and Development Program (ALCA) , Japan Science and Technology Agency (JST) , 4-1-8 Honcho , Kawaguchi , 332-0012 , Japan

## Abstract

Acid–base solid catalysts synthesized with structurally controlled uniform active sites can lead to unique catalysis. In this study, a CePO_4_ catalyst was synthesized using a hydrothermal method and found to exhibit high catalytic performance for the chemoselective acetalization of 5-hydroxymethylfurfural with alcohols, in sharp contrast to other homogeneous and heterogeneous acid and/or base catalysts. In the presence of CePO_4_, various combinations of carbonyl compounds and alcohols are efficiently converted into the corresponding acetal derivatives in good to excellent yields. Mechanistic studies show that CePO_4_ most likely acts as a bifunctional catalyst through the interaction of uniform Lewis acid and weak base sites with 5-hydroxymethylfurfural and alcohol molecules, respectively, which results in high catalytic performance.

## Abstract 2

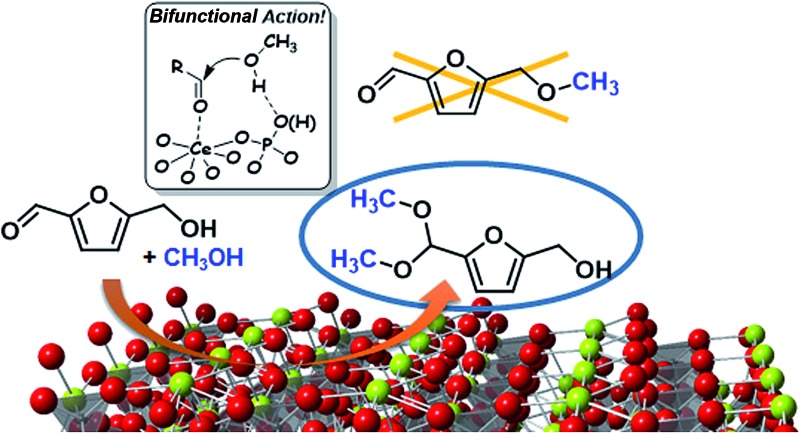
A CePO_4_ catalyst exhibits high catalytic performance for the chemoselective acetalization of 5-hydroxymethylfurfural with alcohols, in sharp contrast to other homogeneous and heterogeneous acid and/or base catalysts.

## Introduction

Synergistic and cooperative activation by two or more catalytically active sites with acidic, basic or redox properties can allow high catalytic activity and specific selectivity.^1^ In particular, acid–base bifunctionality has received much attention because such concepts are widely applied to catalyses related to hydrocarbon conversion, atom-efficient functional group transformation, tandem reactions, and asymmetric syntheses.^2^ In the fields of heterogeneous catalysis, the acid–base properties of metal oxide-based materials have been extensively studied, and various effective simple and mixed oxide catalysts have been reported.^3^ However, difficulty in the construction of uniform electrically and structurally controlled acid–base sites often leads to a problem where the fine-tuning of the catalyst structure and the reactivity are restrained. While the modification of oxide surfaces with organic acids and/or bases is a powerful method,^4^ the susceptibility of the organic parts to oxidative/thermal degradation has limited the usefulness of such catalysts. Therefore, the design and development of new high-performance all-inorganic heterogeneous acid–base catalysts remains a strongly desired and challenging subject of research.

**Fig. 1:**
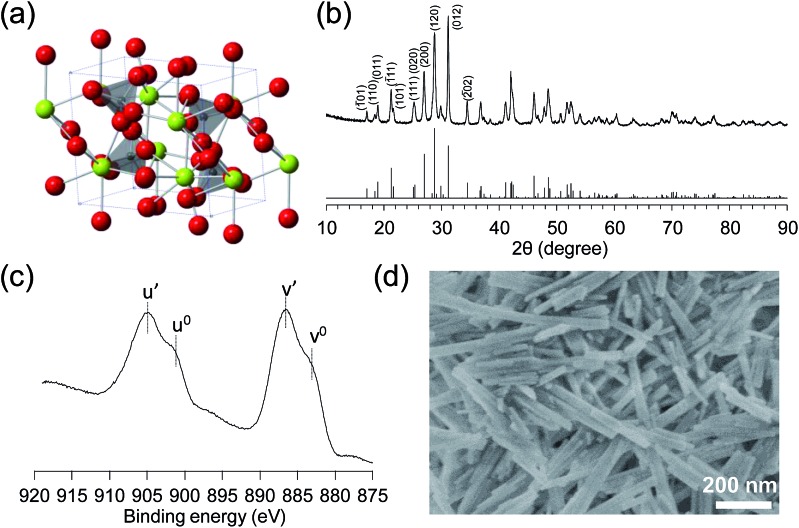
(a) Structure of CePO_4_. Green, gray, and red spheres represent Ce, P, and O atoms, respectively. (b) XRD patterns for CePO_4_ (upper) and monoclinic CePO_4_ (lower, ICSD 79748). (c) XPS Ce 3d spectrum and (d) SEM image of CePO_4_.

We have recently reported unique base catalysis using oxoanions including [WO_4_]^2–^ and [PO_4_]^3–^.^5^ Despite their basicities being weaker than those of inorganic and organic strong bases, their specific activation of nucleophiles such as alcohols and amines results in atom-efficient reactions such as the chemical fixation of CO_2_, regioselective *N*-alkylation of indoles, and chemoselective acylation of alcohols. On the other hand, rare earth (RE) metal species act as Lewis acid catalysts for various carbon–carbon bond forming reactions through the activation of carbonyl compounds.^6^ Against this background, we anticipated that RE orthophosphates, REPO_4_, would be good candidates as bifunctional acid–base catalysts that can work in concert to promote electrophilicity and nucleophilicity in reactive partners. In this communication, we report the highly chemoselective acetalization of 5-hydroxymethylfurfural (**1a**), which has alcohol and aldehyde functionalities,^7^ with alcohols using a monoclinic CePO_4_ catalyst synthesized by a hydrothermal method (Fig. 1(a)). Compound **1a** is a versatile carbonyl compound with sensitive functional groups. For reactions of **1a** with alcohols in the presence of acid catalysts,^8,9^ ethers or a complex mixture of products are typically obtained due to the presence of the Brønsted acid-sensitive hydroxyl groups in **1a**.^8^ The present system has the following significant advantages: (i) high yields and chemoselectivity toward acetals, even for substrates with hydroxyl groups, (ii) applicability to various combinations of substrates and larger-scale syntheses, and (iii) reusability as a heterogeneous catalyst system. While metal phosphates and related materials have been extensively investigated for the conversion of carbohydrates into **1a**,^10^ acid–base catalysis over CePO_4_ has not been reported to date^11^ and the present bifunctionality can lead to the chemoselective acetalization of carbonyl compounds containing sensitive functional groups, such as **1a**.

## Results and discussion

### Synthesis and characterization of CePO_4_

Monoclinic CePO_4_ was synthesized through the hydrothermal reaction of Ce(NO_3_)_3_ and (NH_4_)_2_HPO_4_ at 180 °C, followed by calcination at 900 °C (see details in the ESI†). Fig. 1(b) shows the powder X-ray diffraction (XRD) pattern measured for CePO_4_, which is in good agreement with that reported for the monoclinic CePO_4_ structure, in which a Ce^3+^ ion connects to seven tetrahedral PO_4_^3–^ groups [space group *P*2_1_/*n*].^12^ The impurity phases of other cerium and phosphorus oxides were not observed. The infrared (IR) spectrum of CePO_4_ is shown in Fig. S1.† Asymmetric stretching vibrations of the PO_4_^3–^ groups were split into bands at 1091, 1062, 1028, 994, and 956 cm^–1^ due to a decrease in the symmetry of PO_4_^3–^ from *T*_d_ to *C*_1_.^13^ The bands in the range of 500–700 cm^–1^ are assigned to the bending of the P–O links in the distorted PO_4_^3–^ tetrahedra, and these band positions are similar to those previously reported for monoclinic CePO_4_.^13^ The elemental analysis of bulk CePO_4_ using energy dispersive X-ray spectroscopy (EDX) revealed that the molar ratio of Ce : P is 1 : 1. The valence state of the surface Ce was investigated using X-ray photoelectron spectroscopy (XPS) (Fig. 1(c)). The Ce 3d_3/2,5/2_ spectra are composed of two multiplets (v and u), which correspond to the spin–orbit split 3d_5/2_ and 3d_3/2_ core holes.^14^ The Ce 3d spectrum of CePO_4_ exhibits four peaks with binding energies of 904.9, 901.0, 806.5, and 883.0 eV, which correspond to the u′, u^0^, v′, and v^0^ peaks, respectively, and are in good agreement with the reported Ce 3d spectra for Ce(iii) oxides.^15^ The specific surface area of CePO_4_ calculated from a Brunauer–Emmett–Teller (BET) plot of the N_2_ adsorption isotherm (77 K) was up to 37 m^2^ g^–1^. Fig. 1(d) shows a scanning electron microscopy (SEM) image of CePO_4_ with rod-like shaped particles 100–500 nm long and 20–50 nm wide.

**Fig. 2:**
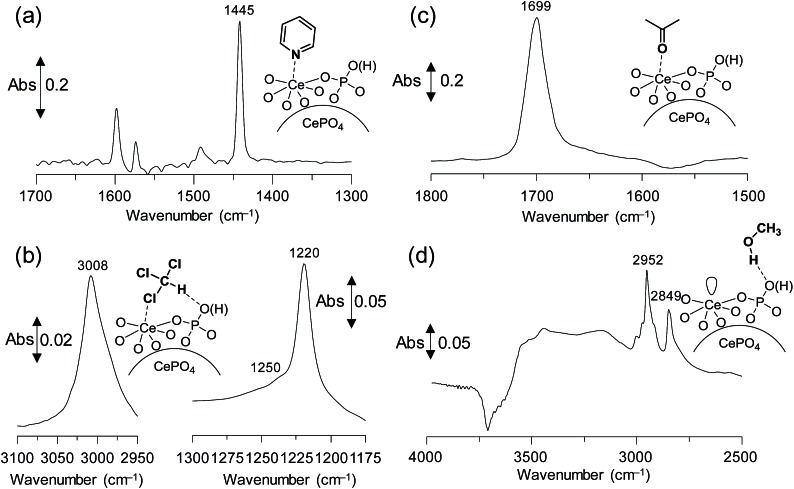
Difference IR spectra for (a) pyridine-, (b) chloroform-, (c) acetone-, and (d) methanol-adsorbed CePO_4_ at 25 °C.

The acidic properties of CePO_4_ were evaluated using IR spectroscopy for a sample with adsorbed pyridine as a probe base (see details in the ESI†).^3,10,16^ Differential IR spectra of CePO_4_ with adsorbed pyridine are shown in Fig. 2(a) and S2.† The band at 1445 cm^–1^ is assigned to the pyridine species coordinated to the Lewis acid sites, while no band due to pyridinium ions bonded to the Brønsted acid sites was observed at *ca.* 1540 cm^–1^. The amount of Lewis acid sites on CePO_4_ was estimated from the intensity of the band at 1445 cm^–1^ to be 0.096 mmol g^–1^. The difference IR spectrum for CePO_4_ with adsorbed CHCl_3_ is shown in Fig. 2(b). The red-shift of the original C–H stretching mode of the CHCl_3_ molecule (from 3034 cm^–1^ to 3008 cm^–1^) indicates the presence of basic sites on the surface.^16^ In addition, a new broad shoulder band appeared at 1250 cm^–1^, which was assigned as *δ*(ClC–H) for the CHCl_3_ molecules due to the interaction of the acidic hydrogen and chlorine atoms with the basic oxygen and Lewis acid sites, respectively.^16^ Thus, the base sites on CePO_4_ could be located in close proximity to the Lewis acid sites, in agreement with the structure of CePO_4_.

### Catalytic acetalization of **1a** with methanol

The reaction of **1a** with methanol was examined first in the presence of various catalysts that have been reported to be effective for acetalization,^17^ and the results are summarized in Table 1. The three products 5-(dimethoxymethyl)-2-furanmethanol (**2a**), 5-methoxymethylfurfural (**3a**), and 2-(dimethoxymethyl)-5-(methoxymethyl)furan (**4a**) were mainly formed. The reaction did not proceed in the absence of a catalyst (entry 27). Among the catalysts tested, CePO_4_ exhibited the highest activity for the acetalization of **1a** to give **2a** in 78% yield (entry 1). In this case, the selectivity toward **2a** reached 96% without the formation of **3a** or **4a**. In the presence of homogeneous Brønsted acid catalysts (H_2_SO_4_, *p*-toluenesulfonic acid (TsOH), and H_3_PW_12_O_40_) and Lewis acid catalysts (scandium trifluoromethanesulfonate (Sc(OTf)_3_) and Ce(OTf)_3_), only **3a** and/or **4a** were obtained in low to moderate yields (entries 2–6). The reaction of **1a** with methanol in the presence of these homogeneous acid catalysts was carried out by reducing the amounts of the catalysts to match the surface Ce content (*i.e.* 9.6 μmol) with the Lewis acid sites measured using pyridine-IR (Table S1†). While the conversions using these homogeneous acid catalysts were higher than that using CePO_4_, **2a** was not formed under the reaction conditions. In addition, no product was obtained using the homogeneous base catalysts of K_3_PO_4_, K_2_HPO_4_, and KH_2_PO_4_ (entries 7–9). Thus, homogeneous acid or base catalysts themselves are not deemed as effective for the chemoselective acetalization of **1a**. Acetalization over Nb_2_O_5_ was less effective than over CePO_4_, and other metal oxide catalysts including SiO_2_, ZrO_2_, CeO_2_, Al_2_O_3_, MgO, TiO_2_, and SnO_2_ were almost inactive (entries 13–20). Typical solid acid catalysts such as sulfated zirconia, sulfonated carbon, Nafion, mordenite, and montmorillonite gave complex mixtures of **2a**, **3a**, and **4a** (entries 21–26). The catalyst precursors of Ce(NO_3_)_3_, (NH_4_)_2_HPO_4_, and a mixture of Ce(NO_3_)_3_ and (NH_4_)_2_HPO_4_ were not effective for acetalization (entries 10–12), which indicates that CePO_4_ plays an important role in the acetalization reaction.^18^

**Table 1:** Effect of catalysts on the reaction of **1a** with methanol^*a*^

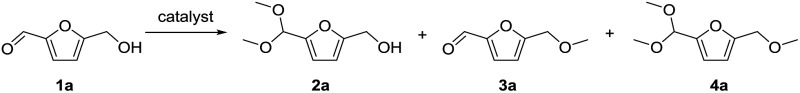					
Entry	Catalyst	Conv. (%)	Yield (%)		
			**2a**	**3a**	**4a**
1	CePO_4_	81	78	<1	<1
2^*b*^	H_2_SO_4_	>99	<1	24	2
3^*b*^	TsOH	>99	<1	54	2
4^*b*^	H_3_PW_12_O_40_	>99	<1	4	<1
5^*b*^	Sc(OTf)_3_	>99	<1	49	5
6^*b*^	Ce(OTf)_3_	74	<1	27	<1
7	K_3_PO_4_	81	<1	<1	<1
8	K_2_HPO_4_	<1	<1	<1	<1
9	KH_2_PO_4_	1	<1	<1	<1
10^*b*^	Ce(NO_3_)_3_·6H_2_O	68	<1	12	<1
11^*b*^	(NH_4_)_2_HPO_4_	43	<1	<1	<1
12^*b*^	Ce(NO_3_)_3_·6H_2_O + (NH_4_)_2_HPO_4_	85	<1	<1	<1
13	Nb_2_O_5_	38	31	<1	<1
14	SiO_2_	12	1	<1	<1
15	ZrO_2_	9	9	<1	<1
16	CeO_2_	5	<1	<1	<1
17	Al_2_O_3_	3	3	<1	<1
18	MgO	3	<1	<1	<1
19	TiO_2_	2	3	<1	<1
20	SnO_2_	2	1	<1	<1
21	Sulfated zirconia	>99	<1	2	15
22	Sulfonated carbon	83	9	15	43
23	Nafion NR-50	95	1	42	21
24	Nafion SAC-13	90	20	2	13
25	Mordenite	86	39	9	43
26	Montmorillonite K10	85	55	4	23
27	Blank	2	2	<1	<1

^*a*^Reaction conditions: catalyst (0.1 g), **1a** (1.0 mmol), methanol (5 mL), and reflux for 1 h. Conversion and yield were determined by GC analysis. Conversion (%) = converted **1a** (mol)/initial **1a** (mol) × 100. Yield (%) = product (mol)/initial **1a** (mol) × 100.

^*b*^Catalyst (0.43 mmol; *i.e.* equivalent to the Ce content in CePO_4_ (0.1 g)).

**Fig. 3:**
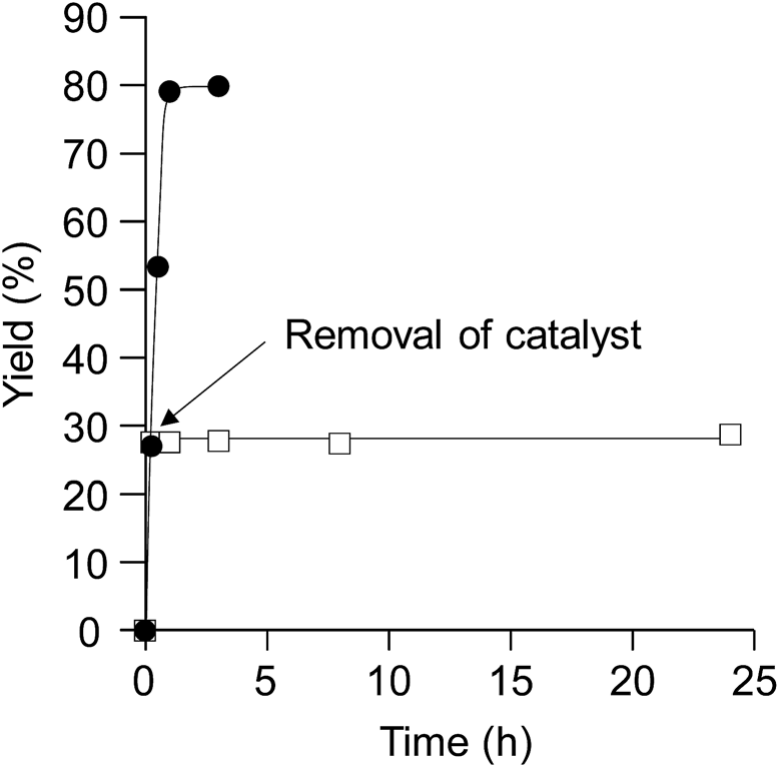
Effect of CePO_4_ removal on the acetalization of **1a** with methanol. Without removal of CePO_4_ (), and the arrow indicates the point of CePO_4_ removal (□). Reaction conditions: CePO_4_ (0.1 g), **1a** (1.0 mmol), methanol (5 mL), and reflux.

To verify whether the observed catalysis is derived from solid CePO_4_ or from leached cerium or phosphorus species, the reaction of **1a** with methanol was conducted under the conditions described in entry 1 of Table 1, and CePO_4_ was removed from the reaction mixture by hot filtration at *ca.* 30% conversion of **1a** (at *t* = 15 min). The filtrate was then heated again under the same reaction conditions. In this case, no further production of **2a** was observed, as shown in Fig. 3. No leaching of cerium or phosphorus species into the filtrate was observed using inductively coupled plasma atomic emission spectroscopy (ICP-AES, with detection limits for Ce and P atoms of *ca.* 1 and 3 ppb, respectively). Therefore, there was no contribution to the observed catalysis from cerium or phosphorus species leached into the reaction solution, and the nature of the observed catalysis is confirmed as truly heterogeneous.^19^ The used CePO_4_ catalyst could be readily recovered from the reaction mixture by simple filtration (96% recovery). The recovered CePO_4_ catalyst could then be reused without a significant decrease in the yield of **2a** or the selectivity: 78% yield of **2a** at 81% conversion (fresh) and 78% yield of **2a** at 80% conversion (reused). There was no significant difference in the XRD patterns of the fresh and reused CePO_4_ catalysts, which indicates the high durability of CePO_4_ (Fig. S3†).

### Reaction mechanism for the CePO_4_-catalyzed acetalization

While it has been reported that other metal oxides (*e.g.* CeO_2_) can function as effective acid–base catalysts,^3,17d^ only CePO_4_ exhibited high catalytic activity and chemoselectivity for acetalization. Thus, the activation mode of substrates with CePO_4_ and CeO_2_ was confirmed using IR measurements of samples with adsorbed acetone and methanol. As shown in Fig. 2(c), one strong C=O stretching band of acetone adsorbed on CePO_4_ was observed at a lower wavenumber (1699 cm^–1^) than that of acetone in the gas phase (1731 cm^–1^).^20^ In addition, the IR spectrum of acetone adsorbed on CeO_2_ exhibited a shoulder at 1700 cm^–1^ and a strong band at 1673 cm^–1^ due to acetone molecules coordinated to different types of Lewis acid sites, and absorptions at 1627 cm^–1^ and 1570–1550 cm^–1^ are assignable to condensed species (Fig. S4†).^20^ These results indicate the interaction between the carbonyl oxygen of the ketone and the uniform Lewis acid sites on CePO_4_ without the promotion of aldol condensation.^21,22^

**Fig. 4:**
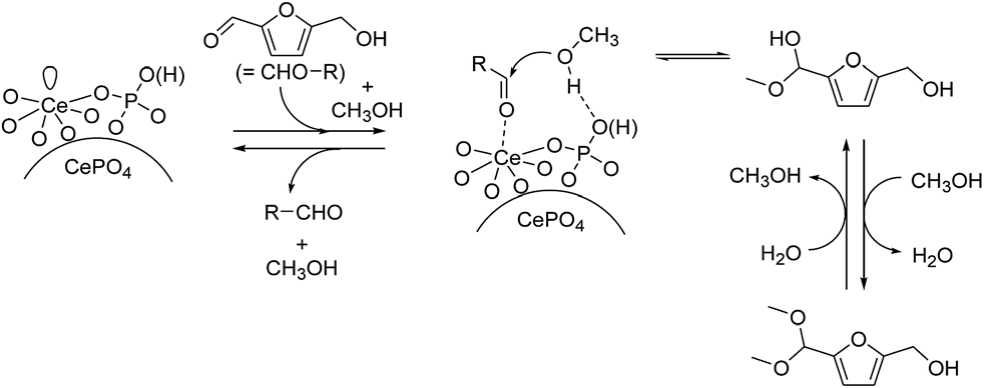
Proposed reaction mechanism for the CePO_4_-catalyzed acetalization of **1a** with methanol.

Fig. 2(d) shows the IR spectrum of methanol adsorbed on CePO_4_. In the *ν*(O–H) region, negative OH bands were observed with the appearance of broad bands between 3000 and 3500 cm^–1^. In addition, the IR spectrum shows bands at 2952 and 2849 cm^–1^ that were assigned to asymmetric and symmetric CH_3_ stretching modes, respectively. The appearance of such broad bands and the band positions of *ν*(CH_3_) indicate that methanol is adsorbed molecularly on CePO_4_ *via* hydrogen bonds, which is consistent with previous reports for the non-dissociative adsorption of methanol on metal oxides.^23^ On the other hand, the IR spectrum for methanol adsorbed on CeO_2_ has bands at 2911 and 2805 cm^–1^ that were assigned to the *ν*_as_(CH_3_) and *ν*_s_(CH_3_) modes of methoxide species, respectively (Fig. S4†). Therefore, CePO_4_ most likely acts as a bifunctional catalyst through interaction of the uniform Lewis acid sites and weak base sites with **1a** and alcohol molecules, respectively, which results in highly efficient and chemoselective acetalization.^24^ The present acetalization of **1a** with methanol possibly proceeds as follows (Fig. 4). First, the activation of both **1a** and methanol by CePO_4_ facilitates nucleophilic attack of the OH group in methanol on the carbon atom of the carbonyl group in **1a** to give the corresponding hemiacetal derivative. Further reaction of the hemiacetal with methanol then occurs, most likely with the assistance of the CePO_4_ catalyst, to give the corresponding acetal derivative.

### Substrate scope for the CePO_4_-catalyzed acetalization

**Fig. 5:**
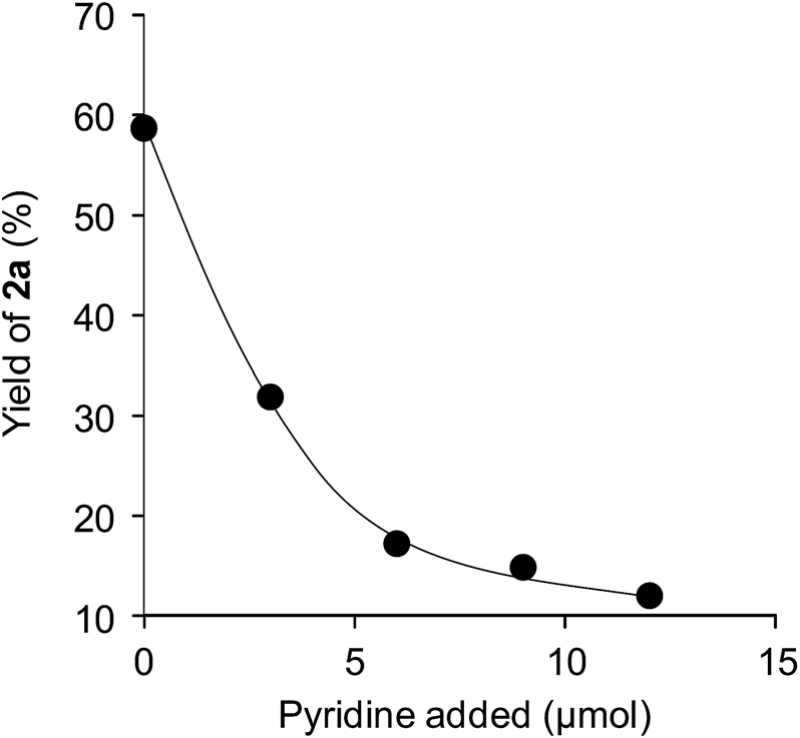
Yields of **2a** against the amount of pyridine added. Reaction conditions: CePO_4_ (0.1 g), **1a** (1.0 mmol), methanol (5 mL), and reflux for 30 min.

To investigate the effectiveness of the bifunctional properties of CePO_4_, CePO_4_-catalyzed acetalization was explored with various substrates. In the presence of CePO_4_, various combinations of carbonyl compounds and alcohols were efficiently converted into the corresponding acetal derivatives in good to excellent yields (Table 2). The acetalization of **1a** with methanol and with diols such as ethylene glycol and 1,3-propanediol proceeded selectively (entries 1–3). CePO_4_ effectively catalyzed the acetalization of other aldehydes containing heteroatoms, such as furfural (**1b**) and 2-thiophenecarboxaldehyde (**1c**), into the corresponding dimethyl acetals, while the acetalization of 2-pyridinecarboxaldehyde (**1d**) did not proceed (entries 4–6). The catalytic reactivity of CePO_4_ in the presence of pyridine was investigated. It was confirmed that the presence of pyridine strongly inhibited the reaction of **1a** with methanol (Fig. 5). The yield of **2a** decreased with an increase in the small amount of pyridine added (3–12 μmol; *ca.* 0.3–1.3 equivalents with respect to the Lewis acid sites on CePO_4_). The nitrogen atom of pyridine would strongly coordinate to the cerium metal center, which would inhibit the reaction. The reactions of benzaldehydes with electron-donating and electron-withdrawing *para*-substituents (**1e–1g**) proceeded to afford the corresponding dimethyl acetals in high yields (entries 7–9). Even the bulkier aldehyde 1-naphthaldehyde (**1h**) could be efficiently acetalized when the reaction time was prolonged to 20 h (entry 10). The acetalization of cinnamaldehyde (**1i**) with methanol proceeded smoothly without influence on the C=C double bond (entry 11). Not only were aromatic and α,β-unsaturated aldehydes converted but also 3-phenylpropionaldehyde (**1j**) and cyclohexanecarboxaldehyde (**1k**) were efficiently converted into the corresponding acetals in high yields (entries 12 and 13). Furthermore, the present system could effectively catalyze aliphatic and aromatic ketones with ethylene glycol. Cyclohexanone (**1l**) was quantitatively converted into 2,2-pentamethylene-1,3-dioxolane (**2l**), and acetophenone (**1m**) gave its corresponding ketal (**2m**) in moderate yield (entries 14 and 15). Even in the presence of hydroxyl groups in the substrate (5-hydroxy-2-adamantanone (**1n**)), the corresponding ketal (**2n**) was obtained in good yield (entry 16).

**Table 2:** Acetalization of carbonyl compounds with alcohols catalyzed by CePO_4_ ^*a*^

Entry	Carbonyl compound	Alcohol	Time (h)	Product (yield (%))
1	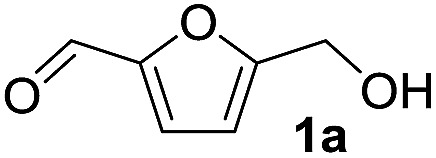	CH_3_OH	1	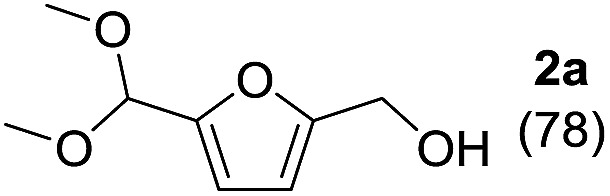
2^*b*^	**1a**	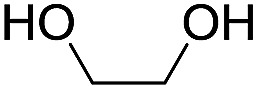	1	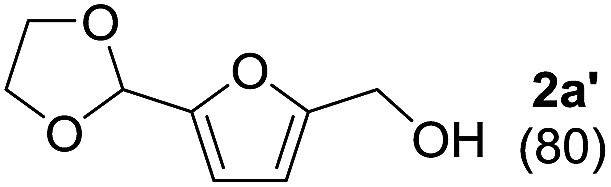
3^*b*^	**1a**	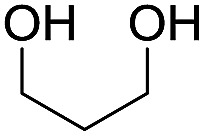	2	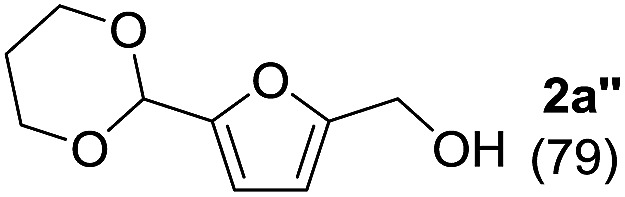
4	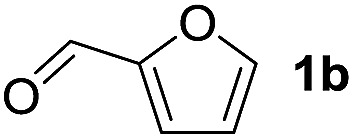	CH_3_OH	6	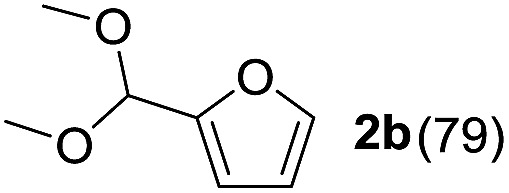
5	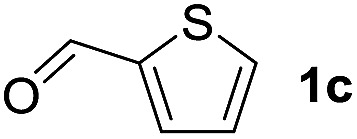	CH_3_OH	6	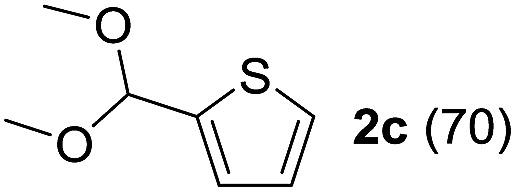
6	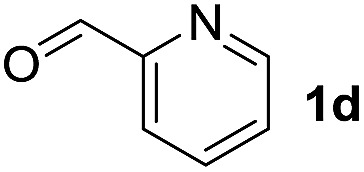	CH_3_OH	6	Not detected
7	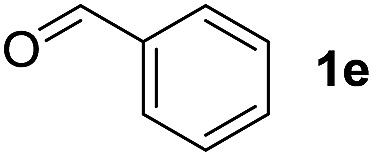	CH_3_OH	6	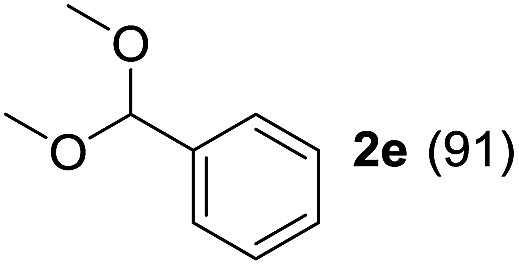
8	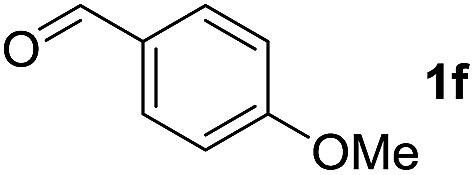	CH_3_OH	6	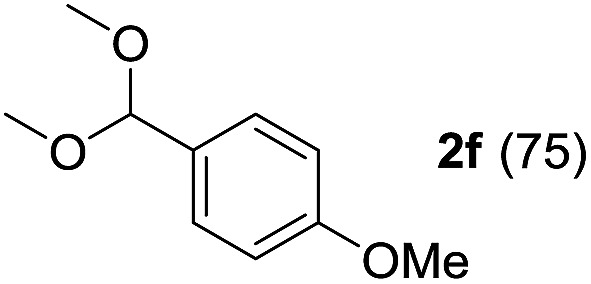
9	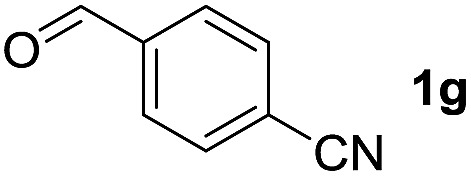	CH_3_OH	6	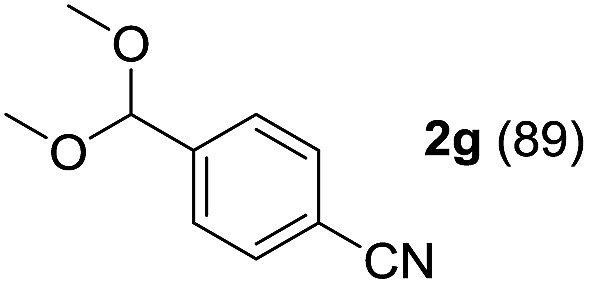
10	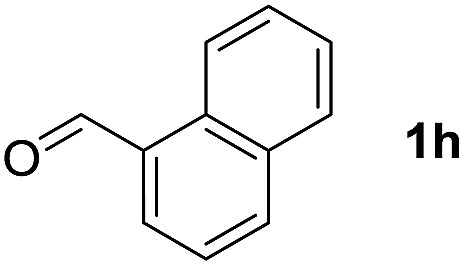	CH_3_OH	20	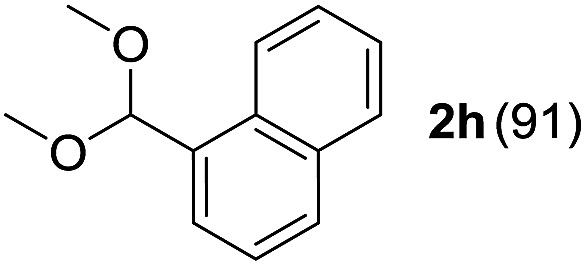
11	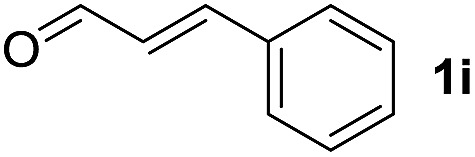	CH_3_OH	6	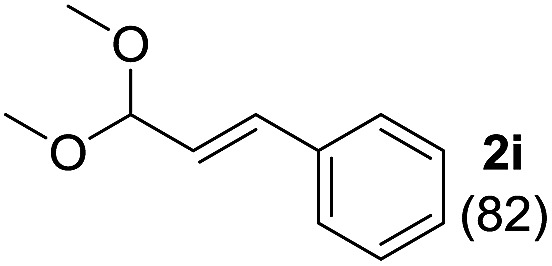
12	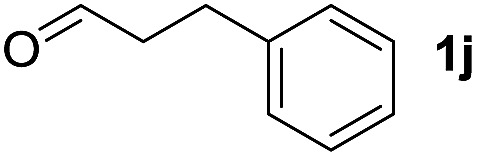	CH_3_OH	20	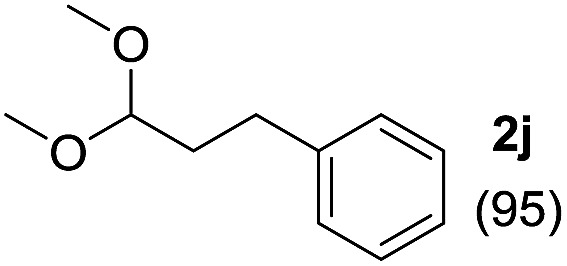
13	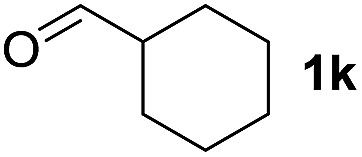	CH_3_OH	20	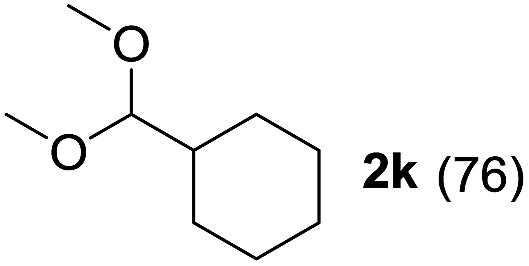
14^*c*^	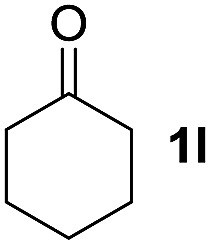	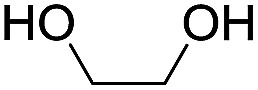	6	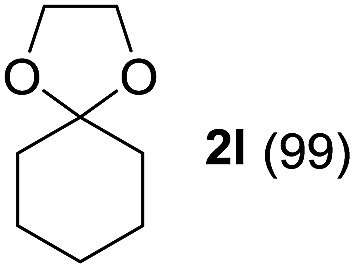
15	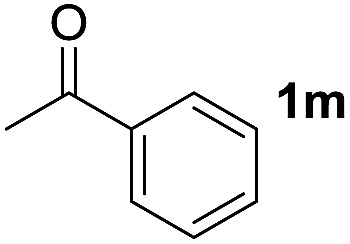	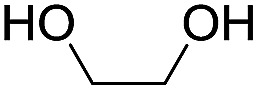	6	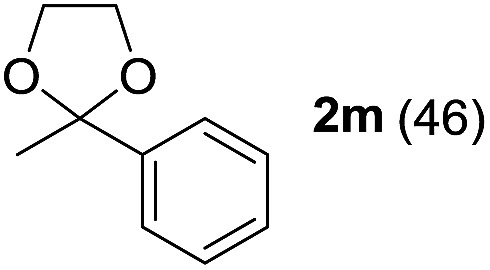
16	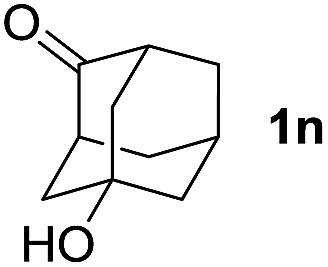	CH_3_OH	6	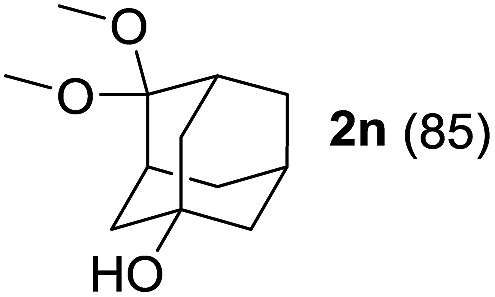

^*a*^Reaction conditions: CePO_4_ (0.1 g), **1** (1.0 mmol), alcohol (5 mL), and reflux. Yields were isolated yields.

^*b*^Yield determined using nuclear magnetic resonance spectroscopy (NMR).

^*c*^120 °C.

The present catalytic system was applicable to a gram-scale reaction of **1a** (10.5 mmol scale) with methanol and 1.46 g of analytically pure **2a** could be isolated (eqn (1)). In this case, the turnover number (TON) based on surface Lewis acid sites reached 177 and the corresponding turnover frequency (TOF) was 44 h^–1^. In addition, CePO_4_ efficiently catalyzed the gram-scale regioselective acetalization of acetone (**1o**) with glycerol into the industrially important chemical 2,2-dimethyl-1,3-dioxolan-5-ol (solketal (**2o**)).^25,26^ Solketal has been used as a highly soluble additive to increase the octane number of fuel, and as such many catalyst systems to aid its synthesis have been reported.^25,26^ CePO_4_ exhibited high regioselectivity (**2o**/**2o′** = 98/2) and 1.29 g of analytically pure **2o** was successfully isolated (eqn (2)), while the condensation of glycerol with **1o** under acidic conditions sometimes affords a mixture of five- and six-membered acetals (**2o** and **2o′**, respectively).^25^

1

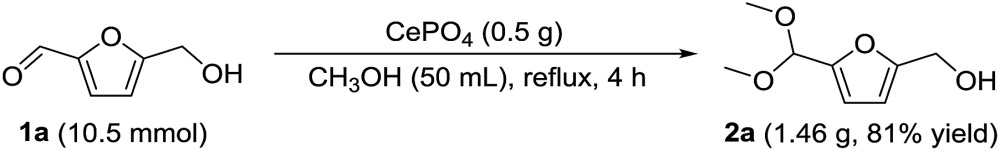



2

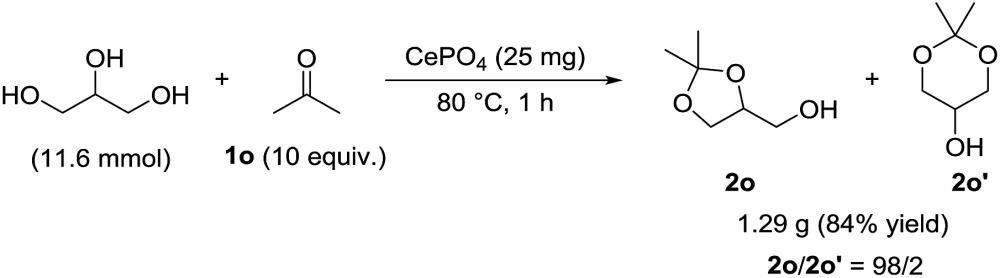




## Conclusions

In conclusion, CePO_4_ efficiently catalyzes the acetalization of various aryl and aliphatic carbonyl compounds containing hydroxyl groups, C=C bonds, and heteroatoms with alcohols. This study suggests that the development of bifunctional solid catalysts with uniform active sites is of particular importance. This approach is a promising strategy for the development of highly efficient heterogeneously-catalyzed reactions through the non-dissociative activation of both electrophiles and nucleophiles under very mild conditions.

## References

[cit1] Topics in Organometallics Chemistry—Bifunctional Molecular Catalysis, ed. T. Ikariya, and M. Shibasaki, Springer, Berlin, 2011.

[cit2] Iglesia E., Barton D. G., Biscardi J. A., Gines M. J. L., Soled S. L. (1997). Catal. Today.

[cit3] (a) Sheldon R. A., Arends I. W. C. E., and Hanefeld U., in Green Chemistry and Catalysis, ed. R. A. Sheldon, I. W. C. E. Arends and U. Hanefeld, Wiley-VCH, Weinheim, 2007, pp. 133–221.

[cit4] Motokura K., Tada M., Iwasawa Y. (2008). Chem.–Asian J..

[cit5] Kimura T., Kamata K., Mizuno N. (2012). Angew. Chem., Int. Ed..

[cit6] Kobayashi S., Manabe K. (2002). Acc. Chem. Res..

[cit7] Rosatella A. A., Simeonov S. P., Frade R. F. M., Afonso C. A. M. (2011). Green Chem..

[cit8] Casanova O., Iborra S., Corma A. (2010). J. Catal..

[cit9] Arias K. S., Al-Resayes S. I., Climent M. J., Corma A., Iborra S. (2013). ChemSusChem.

[cit10] Nakajima K., Baba Y., Noma R., Kitano M., Kondo J. N., Hayashi S., Hara M. (2011). J. Am. Chem. Soc..

[cit11] Yao W., Liu Y., Wang X., Weng X., Wang H., Wu Z. (2016). J. Phys. Chem. C.

[cit12] Bao J., Yu R., Zhang J., Yang X., Wang D., Deng J., Chen J., Xing X. (2009). CrystEngComm.

[cit13] Wang K., Zhang J., Wang J., Fang C., Yu W., Zhao X., Xu H. (2005). J. Appl. Crystallogr..

[cit14] Beche E., Charvin P., Perarnau D., Abanades S., Flamant G. (2008). Surf. Interface Anal..

[cit15] Mullins D. R., Overbury S. H., Huntley D. R. (1998). Surf. Sci..

[cit16] Komanoya T., Nakajima K., Kitano M., Hara M. (2015). J. Phys. Chem. C.

[cit17] Izumi Y., Urabe K., Onaka M. (1998). Microporous Mesoporous Mater..

[cit18] The acetalization of **1a** with methanol in the presence of CePO_4_ was carried out under the same reaction conditions as those for beta-zeolite reported in ref. 9. The **2a** yield was 6% and lower than that of calcined and dehydrated Al-beta zeolite (*ca.* 600 m^2^ g^–1^, Si/Al = 12.5), whereas the reactivity per unit surface area was comparable to that of zeolite

[cit19] Sheldon R. A., Wallau M., Arends I. W. C. E., Schuchardt U. (1998). Acc. Chem. Res..

[cit20] Zaki M. I., Hasan M. A., Al-Sagheer F. A., Pasupulety L. (2000). Langmuir.

[cit21] The amount of Ce cations exposed on the surface was estimated assuming that the (011) plane is a possible surface structure because of its stability.22 The amount of surface Ce cations was estimated to be 3.1 nm^–2^. This value was almost comparable to that (1.6 nm^–2^) calculated from the BET surface area of CePO_4_ (37 m^2^ g^–1^) and the surface Ce cations with Lewis acid sites measured using pyridine-IR (0.096 mmol g^–1^). These results also support the idea that uniform Lewis acid sites exist on CePO_4_

[cit22] Zhang Y., Guan H. (2003). J. Cryst. Growth.

[cit23] Martinez-Ramirez Z., Flores-Escamilla G. A., Berumen-España G. S., Jimenez-Lam S. A., Handy B. E., Cardenas-Galindo M. G., Sarmiento-Lopez A. G., Fierro-Gonzalez J. C. (2015). Appl. Catal., A.

[cit24] The C=O stretching band (1699 cm^–1^) of acetone adsorbed on CePO_4_ was observed at a higher wavenumber than that on CeO_2_ (a strong band at 1673 cm^–1^), indicating the lower Lewis acid strength of CePO_4_. In addition, the chloroform- and methanol-adsorbed IR measurements also indicate the lower basicity of CePO_4_. Therefore, not only the presence of moderate Lewis acid sites but also the weakening of the basicity by replacement of the strong basic sites of CeO_2_ using PO_4_ would suppress side reactions such as aldol condensation, resulting in the present high chemoselectivity

[cit25] Khayoon M. S., Hameed B. H. (2013). Appl. Catal., A.

[cit26] Souza T. E., Portilho M. F., Souza P. M. T. G., Souza P. P., Oliveira L. C. A. (2014). ChemCatChem.

