# Supplementary material for: A bifunctional cerium phosphate catalyst for chemoselective acetalization
Source: Chem Sci. 2017 Feb 7;8(4):3146–53. doi: 10.1039/c6sc05642c (PMC5413973; doi:10.1039/c6sc05642c)
Supplement: Supplementary file 1 [file SC-008-C6SC05642C-s001.pdf]

Electronic Supplementary Material (ESI) for Chemical Science.
This journal is © The Royal Society of Chemistry 2017

Electronic Supplementary Information (ESI) for

A Bifunctional Cerium Phosphate Catalyst for Chemoselective Acetalization of 5-Hydroxymethylfurfural

Shunsuke Kanai,[1] Ippei Nagahara,[1] Yusuke Kita,[1] Keigo Kamata,[1] and Michikazu Hara[1,2]*

[1] Laboratory for Materials and Structures, Institute of Innovative Research, Tokyo Institute of Technology, Nagatsuta-cho 4259, Midori-ku, Yokohama 226-8503, Japan
[2] Japan Science and Technology Agency (JST), Advanced Low Carbon Technology Research and Development Program (ALCA), 4-1-8 Honcho, Kawaguchi 332-0012, Japan
E-mail: hara.m.ae@m.titech.ac.jp

Experimental Section

Materials. Metal oxides (TiO_2 (ST-01, Ishihara Sangyo Kaisha, Ltd., 119 m^2g^{-1}), ZrO_2 (Sigma-Aldrich, 98 m^2g^{-1}), CeO_2 (Daiichi Kigenso Kagaku Kogyo Co., Ltd., 79 m^2g^{-1}), Nb_2O_5 (CBMM, 110 m^2g^{-1}), Al_2O_3 (JRC-ALO-6, Nikki-Universal Co., Ltd. , 155 m^2g^{-1}), MgO (Ube Material, 35 m^2g^{-1}), SiO_2 (Q-10, FUJI SILYSIA, 250 m^2g^{-1}), SnO_2 (Kanto Chemical, 31 m^2g^{-1})) were pretreated in air at 400 °C for 2 h. Inorganic reagents (sulfonated zirconia (Kujundo Chemical Laboratory Co., Ltd.), H_2SO_4 (Kanto Chemical), TsOH (Kanto Chemical), $H_3PW_{12}O_{40}$ (Wako Chemical), sulfonated carbon (Futamura Chemical Co., Ltd.), Nafion NR-50 (Sigma-Aldrich), Nafion SAC-13 (Sigma-Aldrich), mordenite (Wako Chemical), Monmorillonite K-10 (Sigma-Aldrich), $Sc(OTf)_3$ (TCI Chemical), $Ce(OTf)_3$ (TCI Chemical), K_3PO_4 (Kanto Chemical), $Ce(NO_3)_3{\cdot}6H_2O$ (Kanto Chemical), and $(NH_4)_2HPO_4$ (Wako Chemical)) and organic reagents (5-hydroxymethylfurfural (Sigma-Aldrich), methanol (dehydrated, Kanto Chemical), and other substrates and solvents (Sigma-Aldrich, TCI Chemical, or Kanto Chemical)) were purchased and used as received.

Instruments. X-ray diffraction (XRD) patterns were recorded on a diffractometer (Ultima IV, Rigaku; Cu Kα, λ = 1.5405 Å, 40 kV–40 mA) equipped with a high-speed 1-

dimensional detector (D/teX Ultra, Rigaku). Diffraction data were collected in the range of 2θ = 10–80° in 0.02° steps with a scan rate of 20°/min. Nitrogen adsorption-desorption isotherms were measured at –196 °C with a surface area analyzer (Nova-4200e, Quantachrome). Prior to measurement, the samples were heated at 150 °C for 1 h under vacuum to remove physisorbed water. The Brunauer-Emmett-Teller (BET) surface areas were estimated over the relative pressure (P/P_0) range of 0.05–0.30. CO_2 temperature-programmed desorption analysis was performed with BELCAT-A (BEL Japan). Fourier transform infrared (FTIR) spectra were obtained at a resolution of 4 cm^{-1} using a spectrometer (FT/IR-6100, Jasco) equipped with an extended KBr beam-splitting device and a mercury cadmium telluride (MCT) and a triglycine sulfate (TGS) detectors. Energy dispersive X-ray spectrometry (EDX) was performed with a Shimadzu EDX-7000 spectrometer. Inductively coupled plasma-atomic emission spectroscopy (ICP-AES) analyses were performed with a Shimadzu ICPS-8100 spectrometer. Differential thermal analysis (DTA) and thermogravimetric (TG) measurements were performed with a differential thermal analyzer (TG8120, Rigaku). X-ray photoelectron spectroscopy (XPS; JPC-9010MC, Jeol) was performed using Mg Kα radiation (1253.6 eV) at 10 kV and 25 mA. Samples were pressed into pellets and fixed on double-sided carbon tape. The binding energies were calibrated using the C 1s band at 284.6 eV. The morphology of the samples was examined using scanning electron microscopy (SEM; S-5500, Hitachi). Liquid-phase catalytic oxidation was performed with an organic synthesizer (ALHB-80 & DTC-200HZ-3000, Techno Applications; CHEMIST PLAZA CP-1000, Sibata). Distillation of substrates or drying of samples were performed with a glass oven (B-585 Kugelrohr, BUCHI). The isolation of products was performed with a single channel automated flash chromatography system (Smart Flash EPCLC AI-580S, Yamazen). Nuclear magnetic resonance (NMR) spectra were recorded on a Bruker biospin Avance III spectrometer (^{1}H, 400 MHz; ^{13}C, 100 MHz) using 5 mm tubes. Chemical shifts (δ) were reported in ppm downfield from $SiMe_4$ (solvent, $CDCl_3$). Gas chromatography (GC) analyses were performed on Shimadzu GC-2025 equipped with a Stabilwax capillary column (internal diameter = 0.25 mm, length = 30 m) and with a flame ionization detector, or on Shimadzu GC-17A with an InertCap 17 capillary column (internal diameter = 0.25 mm, length = 30 m). Mass spectra were recorded on a spectrometer (GCMS-QP2010 SE, Shimadzu) equipped with an InertCap 17MS capillary column (internal diameter = 0.25

mm, length = 30 m) at an ionization voltage of 70 eV.

Synthesis of a $CePO_4$ Catalyst. $CePO_4$ was synthesized by the hydrothermal reaction. First, $Ce(NO_3)_3{\cdot}6H_2O$ (3 mmol, 1.30 g) and $(NH_4)_2HPO_4$ (3 mmol, 0.396 g) were dissolved in water (35 mL) and stirred for 1 h at room temperature. The resulting solution was then transferred into a stainless steel autoclave with Teflon vessel liner (TAF-SR type, Taiatsu Techno Corporation). After the solution was heated at 180 ºC for 96 h, the precipitates were collected by filtration and washed with water (150 mL). Then, the resulting precipitates were calcined at 900 ºC for 3 h and the $CePO_4$ catalyst was obtained (0.68 g, 96% yield). In addition, we could successfully increase the synthesis scale by three-fold and synthesize the analytically pure $CePO_4$ (2.05 g, 97% yield).

Procedure for Catalytic Acetalization. The catalytic acetalization of various carbonyl compounds was conducted in a 30 mL glass vessel containing a magnetic stirring bar. A typical procedure for the catalytic acetalization of **1a** was as follows. **1a** (1.0 mmol), $CePO_4$ (0.1 g), methanol (5 mL), and internal standard (naphthalene) were charged into the glass reactor. The reaction solution was heated under the reflux conditions (bath temperature: 80 °C) for 1 h. The reaction solution was periodically analyzed by GC, GC-MS, and NMR. After the reaction was completed, the catalyst was separated by filtration. After the removal of methanol by evaporation, the analytically pure product was then isolated using a flash chromatography separation system with amino-bonded silica gel (pore size 60 Å, particle size 40 μm) and $CHCl_3$/MeOH (for **2a** isolation) or *n*-hexane/ethyl acetate as eluents. The recovered catalyst was washed with methanol (20 mL), evacuated to dryness, and reused.

Effect of Additional Pyridine on the Acetalization of 1a with Methanol. The catalytic reactivity of $CePO_4$ in the presence of pyridine was investigated under the reaction conditions in Figure 5. It was confirmed that the presence of pyridine strongly inhibited the reaction of **1a** with methanol. The yield of **2a** decreased with an increase in the small amount of pyridine added (3–12 μmol; ca. 0.3–1.3 equivalents with respect to the Lewis acid sites on $CePO_4$). The nitrogen atom of pyridine would strongly coordinate to the cerium metal center, which would inhibit the reaction.

A Larger-Scale (10.5 mmol scale) Acetalization of 1a with Methanol. Into a 300 mL glass reactor were successively placed $CePO_4$ (0.5 g), **1a** (10.5 mmol, 1.325 g), and methanol (50 mL). The reaction mixture was stirred under the reflux conditions (bath

temperature: 80 °C) for 4 h. The catalyst was recovered by filtration. Then, methanol was removed by evaporation. The analytically pure **2a** (1.46 g, 81% yield) was isolated as transparent liquids using a flash chromatography separation system.

A Larger-Scale (11.6 mmol scale) Synthesis of Solketal by Acetalization of 1o with Glycerol. Into a 30 mL glass reactor were successively placed $CePO_4$ (0.025 g), glycerol (11.6 mmol, 1.07 g), and **1o** (116 mmol, 8.5 mL). The reaction mixture was stirred at 80 °C for 1 h. The catalyst was recovered by filtration. Then, acetone was removed by evaporation. The analytically pure **2o** (1.29 g, 84% yield) was isolated as transparent liquids using a flash chromatography separation system.

Procedure for IR measurements. The amounts of Lewis and Brønsted acid sites on $CePO_4$ were estimated using FT-IR measurements for pyridine-adsorbed samples at 25 °C. $CePO_4$ sample was pressed into self-supporting disks (20 mm diameter, 41.6 mg) and placed in an IR cell attached to a closed glass-circulation system. Prior to pyridine adsorption, the sample was dehydrated by heating at 200 °C for 1 h under vacuum. The intensities of the band at 1450 cm^{-1} (pyridine coordinatively bonded to Lewis acid sites, molecular absorption coefficient: 0.97 $cm \cdot \mu mol^{-1}$) were plotted against the amounts of pyridine adsorbed on the Lewis acid sites of the sample. The intensities of the band increased with the amount of chemisorbed pyridine, reaching plateaus with the appearance of the band due to physisorbed pyridine (1440 cm^{-1}). While the band at 1440 cm^{-1} disappeared after evacuation at room temperature for 3 h, there was no significant difference in intensity of the band at 1450 cm^{-1} before and after evacuation, which indicated that the maximum intensity of the band at 1450 cm^{-1} correspond to the amounts of Lewis acid sites available to chemisorb pyridine to saturation. The amounts of Lewis acid sites on $CePO_4$ were estimated from the maximum band intensities and molecular absorption coefficient at 1450 cm^{-1}. Similarly, the amounts of Lewis and Brønsted acid sites on CeO_2 were estimated to be 0.12 and 0.02 mmol g^{-1}, respectively. As for the IR measurements for chloroform-adsorbed samples at 25 °C, the dehydrated sample (by heating at 200 °C for 1 h under vacuum) was exposed to chloroform vapor. Gas-phase chloroform pressured were 2.2×10^{-1} and 1.1×10^{-1} kPa for CeO_2 and $CePO_4$ samples, respectively. As for the IR measurements for acetone and methanol-adsorbed samples at 25 °C, the dehydrated sample (by heating at 200 °C for 1 h under vacuum) was exposed to saturated acetone and methanol vapor and then evacuated at 25 ºC for 3 h to remove

weakly physisorbed acetone and methanol.

Data of products

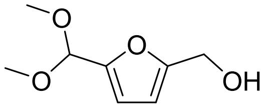

2a

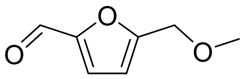

3a

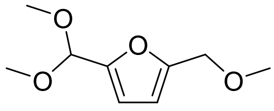

4a

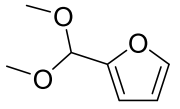

2b

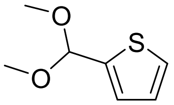

2c

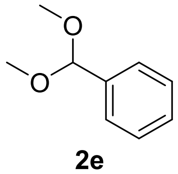

2e

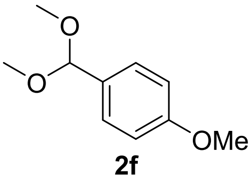

2f

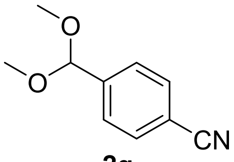

2g

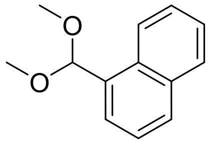

2h

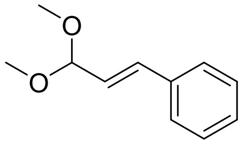

2i

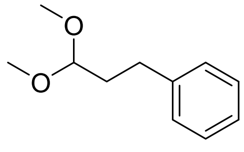

2j

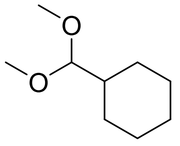

2k

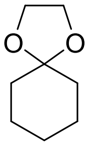

2l

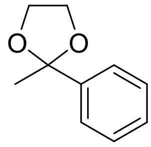

2m

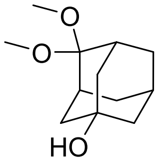

2n

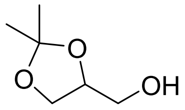

2o

(5-(Dimethoxymethyl)furan-2-yl)methanol (2a)[S1]**:** ^{1}H NMR (400 MHz, $CDCl_3$, 30 °C): 6.34 (d, *J* = 3.1 Hz, 1H), 6.24 (d, *J* = 3.1 Hz, 1H), 5.38 (s, 1H), 4.56 (s, 2H), 3.33 (s, 6H); ^{13}C NMR (100 MHz, $CDCl_3$, 30 °C): 151.8, 151.1, 109.7, 109.1, 66.3, 57.8, 52.8.

5-Methoxymethylfurfural (3a)[S2]**:** ^{1}H NMR (400 MHz, $CDCl_3$, 30 °C): 9.63 (s, 1H), 7.21 (d, *J* = 3.6 Hz, 1H), 6.53 (s, *J* = 3.6 Hz, 1H), 4.49 (s, 2H), 3.43 (s, 3H); ^{13}C NMR (100 MHz, $CDCl_3$, 30 °C): 177.9, 158.5, 152.9, 121.7, 111.3, 66.8, 58.9.

2-(Dimethoxymethyl)-5-(methoxymethyl)furan (4a): ^{1}H NMR (400 MHz, $CDCl_3$, 30 °C): 6.38 (d, *J* = 3.2 Hz, 1H), 6.31 (s, *J* = 3.2 Hz, 1H), 5.42 (s, 1H), 4.39 (s, 2H), 3.36 (s, 9H) ; ^{13}C NMR (100 MHz, $CDCl_3$, 30 °C): 152.0, 151.3, 109.9, 109.3, 98.2, 66.5, 58.0, 53.1.

2-(Dimethoxymethyl)furan (2b)[S3]**:** ^{1}H NMR (400 MHz, $CDCl_3$, 30 °C): 7.41 (dd, *J* = 1.6, 0.8 Hz, 1H), 6.42 (ddd, *J* = 3.2, 0.8, 0.8 Hz, 1H), 6.37 (dd, *J* = 3.2, 1.6 Hz, 1H), 5.44 (d, *J* = 0.8 Hz, 1H), 3.36 (s, 6H); ^{13}C NMR (100 MHz, $CDCl_3$, 30 °C): 150.8, 142.5, 110.0, 108.4, 97.9, 52.8.

2-(Dimethoxymethyl)thiophene (2c)[S4]**:** ^{1}H NMR (400 MHz, $CDCl_3$, 30 °C): 7.30 (dd, *J* = 5.0, 1.2 Hz, 1H), 7.08 (ddd, *J* = 3.6, 1.2, 1.2 Hz, 1H), 7.01 (dd, *J* = 5.0, 3.6 Hz, 1H), 5.64 (d, *J* = 1.2 Hz, 1H), 3.37 (s, 6H); ^{13}C NMR (100 MHz, $CDCl_3$, 30 °C): 141.6, 126.8,

125.7, 125.5, 100.1, 52.5.

Benzaldehyde dimethyl acetal (2e)[S5]**:** ^{1}H NMR (400 MHz, $CDCl_3$, 30 °C): 7.46-7.44 (m, 2H), 7.38–7.32 (m, 3H), 5.39 (s, 1H), 3.33 (s, 6H); ^{13}C NMR (100 MHz, $CDCl_3$, 30 °C): 137.6, 128.0, 127.8, 126.2, 102.7, 52.3.

4-Methoxybenzaldehyde dimethyl acetal (2f)[S6]**:** ^{1}H NMR (400 MHz, $CDCl_3$, 30 °C): 7.35 (d, *J* = 6.8 Hz, 2H), 6.88 (d, *J* = 6.8 Hz, 2H), 5.33 (s, 1H), 3.79 (s, 3H), 3.30 (s, 6H). ^{13}C NMR (100 MHz, $CDCl_3$, 30 °C): 159.7, 130.2, 127.9, 113.5, 103.2, 55.2, 50.4.

4-Cyanobenzaldehyde dimethyl acetal (2g)[S5]: ^{1}H NMR (400 MHz, $CDCl_3$, 30 °C): 7.68 (d, *J* = 8.1 Hz, 2H), 7.58 (d, *J* = 8.1 Hz, 2H), 5.43 (s, 1H), 3.32 (s, 6H); ^{13}C NMR (100 MHz, $CDCl_3$, 30 °C): 143.2, 132.1, 127.6, 118.7, 112.3, 101.8, 52.7.

1-(Dimethoxymethyl)naphthalene (2h)[S7]**:** ^{1}H NMR (400 MHz, $CDCl_3$, 30 °C): 8.30 (d, *J* = 8.1 Hz, 1H), 7.90–7.85 (m, 2H), 7.75 (d, *J* = 7.0 Hz, 1H), 7.56–7.47 (m, 3H), 5.95 (s, 1H), 3.41 (s, 6H); ^{13}C NMR (100 MHz, $CDCl_3$, 30 °C): 133.8, 133.0, 130.8, 129.2, 128.5, 126.2, 125.6, 124.9, 124.8, 124.2, 102.3, 53.2.

***E*-(3,3-dimethoxyprop-1-en-1-yl)benzene (2i)**[S6]**:** ^{1}H NMR (400 MHz, $CDCl_3$, 30 °C): 7.44–7.39 (m, 2H), 7.33–7.31 (m, 2H), 7.29–7.24 (m, 1H), 6.72 (d, *J* = 16.4 Hz, 1H), 6.15 (dd, *J* = 16.4, 5.2 Hz, 1H), 4.96 (d, *J* = 5.2 Hz, 1H), 3.38 (s, 6H); ^{13}C NMR (100 MHz, $CDCl_3$, 30 °C): 136.1, 133.6, 128.6, 128.1, 126.8, 125.7, 103.0, 52.8.

3,3-Dimethoxypropyl)benzene (2j)[S8]**:** ^{1}H NMR (400 MHz, $CDCl_3$, 30 °C): 7.31 (2H), 7.23–7.19 (m, 3H), 4.40 (t, *J* = 5.7 Hz, 1H), 3.36 (s, 6H), 2.71 (t, *J* = 7.6 Hz, 2H), 1.95 (dt, *J* = 7.6, 5.7 Hz, 2H); ^{13}C NMR (100 MHz, $CDCl_3$, 30 °C): 141.6, 128.4 (2C), 125.9, 103.8, 52.7, 34.1, 30.9.

(Dimethoxymethyl)cyclohexane (2k)[S9]**:** ^{1}H NMR (400 MHz, $CDCl_3$, 30 °C): 4.01 (d, *J* = 7.2 Hz, 1H), 3.35 (s, 6H), 1.81–1.74 (m, 4H), 1.69–1.57 (m, 2H), 1.29–1.21 (m, 3H), 1.19–0.95 (m, 2H); ^{13}C NMR (100 MHz, $CDCl_3$, 30 °C): 108.5, 53.5, 40.0, 28.0, 26.3, 25.8.

1,4-Dioxaspiro[4.5]decane (2l)[S8]**:** ^{1}H NMR (400 MHz, $CDCl_3$, 30 °C): 3.94 (s, 4H), 1.61-1.59 (m, 8H), 1.42–1.40 (m, 2H); ^{13}C NMR (100 MHz, $CDCl_3$, 30 °C): 108.9, 64.1, 35.1, 35.1, 23.9.

2-Methyl-2-phenyl-1,3-dioxolane (2m)[S10]**:** ^{1}H NMR (400 MHz, $CDCl_3$, 30 °C): 7.50-7.47 (m, 2H), 7.36-7.27 (m, 3H), 4.06–4.02 (m, 2H), 3.80-3.76 (m, 2H), 1.66 (s, 3H); ^{13}C NMR (100 MHz, $CDCl_3$, 30 °C): 143.5, 128.7, 128.0, 126.0, 109.1, 64.8, 27.7.

5,5-Dimethoxy-2-adamantanone (2n): ^{1}H NMR (400 MHz, $CDCl_3$, 30 °C): 3.16 (s, 3H), 3.15 (s, 3H), 2.21 (s, 2H), 2.08 (s, 1H), 1.94 (d, *J* = 1.2 Hz, 2H), 1.81 (d, *J* = 1.2 Hz, 2H), 1.69 (s, 2H), 1.52 (s, 1H) , 1.49 (s, 2H) , 1.45 (s, 1H) , 1.42 (s, 1H); ^{13}C NMR (100 MHz, $CDCl_3$, 30 °C): 101.2, 68.0, 47.2, 46.8, 45.1, 41.3, 35.1, 32.5, 29.7.

Solketal (2o)[S11]**:** ^{1}H NMR (400 MHz, $CDCl_3$, 30 °C): 4.21–4.28 (m, 1H), 4.04 (dd, J = 8.0, 2.8 Hz, 1H), 3.77-3.83 (m, 1H), 3.74 (dd, J = 7.6, 3.6 Hz, 1H), 3.60 (dd, J = 12.0, 5.2 Hz, 1H), 1.95 (brs, 1H), 145 (s, 3H), 1.38 (s, 3H); ^{13}C NMR (100 MHz, $CDCl_3$, 30 °C): 109.6, 76.2, 65.7, 63.0, 26.7, 25.2.

References

S1 L. Cottier, F. Descotes, and Y. Soro, *J. Carbohyd. Chem.*, 2005, **24**, 55–71

S2 I. Viil, A. Bredihhin, U. Mäeorg, and L. Vares, *RSC Adv.*, 2014, **4**, 5689–5693.

S3 R. Gopinath, Sk. J. Haque, and B. K. Patel, *J. Org. Chem.*, 2002, **67**, 5842–5845.

S4 H. Fujioka, A. Goto, K. Ohtake, O. Kubo, Y. Sawama, and T. Maegawa, *Chem. Commun.*, 2011, **47**, 9894–9896.

S5 M. Baxter, and Y. Bolshan, *Chem. Eur. J.*, 2015, **21**, 13535–13538.

S6 L. Myles, R. G. Gore, N. Gathergood, and S. J. Connon, *Green Chem.*, 2013, **15**, 2740–2746.

S7 M. Zhan, Y. Wang, Y. Yang, and X. Hu, *Adv. Synth. Catal.*, 2012, **354**, 981–985.

S8 A. Ladépêche, E. Tam, J.-E. Ancel, and L. Ghosez, *Synthesis*, 2004, 1375–1380.

S9 L. Gremaud, and A. Lexakis, *Angew. Chem., Int. Ed.*, 2012, **51**, 794–797.

S10 U. Azzena, M. Carraro, A. D. Mamuye, I. Murqia, L. Pisano, and G. Zedde, *Green Chem.*, 2015, **17**, 3281–3284.

S11 D. Pan, J. Sun, H. Jin, Y. Li, L. Li, Y. Wu, L. Zhang, and Z. Yang, *Chem. Commun.*, 2015, **51**, 469–472.

Table S1 Effect of catalysts on the reaction of **1a** with methanol[a]

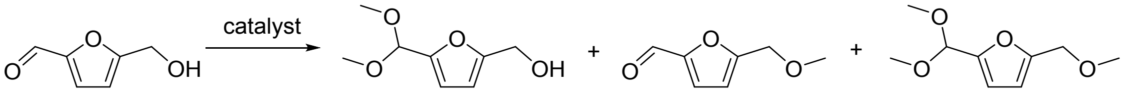

Entry	Catalyst	Conv. (%)	Yield (%)		
			**2a**	**3a**	**4a**
1	$CePO_4$	81	78	<1	<1
2[b]	H_2SO_4	99	<1	63	8
3[b]	TsOH	92	1	16	12
4[b]	$H_3PW_{12}O_{40}$	99	<1	72	6
5[b]	$Sc(OTf)_3$	88	1	32	8
6[b]	$Ce(OTf)_3$	85	3	7	3

[a] Reaction conditions: Catalyst (0.1 g), **1a** (1.0 mmol), methanol (5 mL), reflux, 1 h. Conversion and yield were determined by GC analysis. Conversion (%) = converted **1a** (mol)/initial **1a** (mol) × 100. Yield (%) = product (mol)/initial **1a** (mol) × 100. [b] Catalyst (9.6 μmol; i.e., equivalent to the surface Ce content with Lewis acid sites measured by pyridine-IR on $CePO_4$ (0.1 g)).

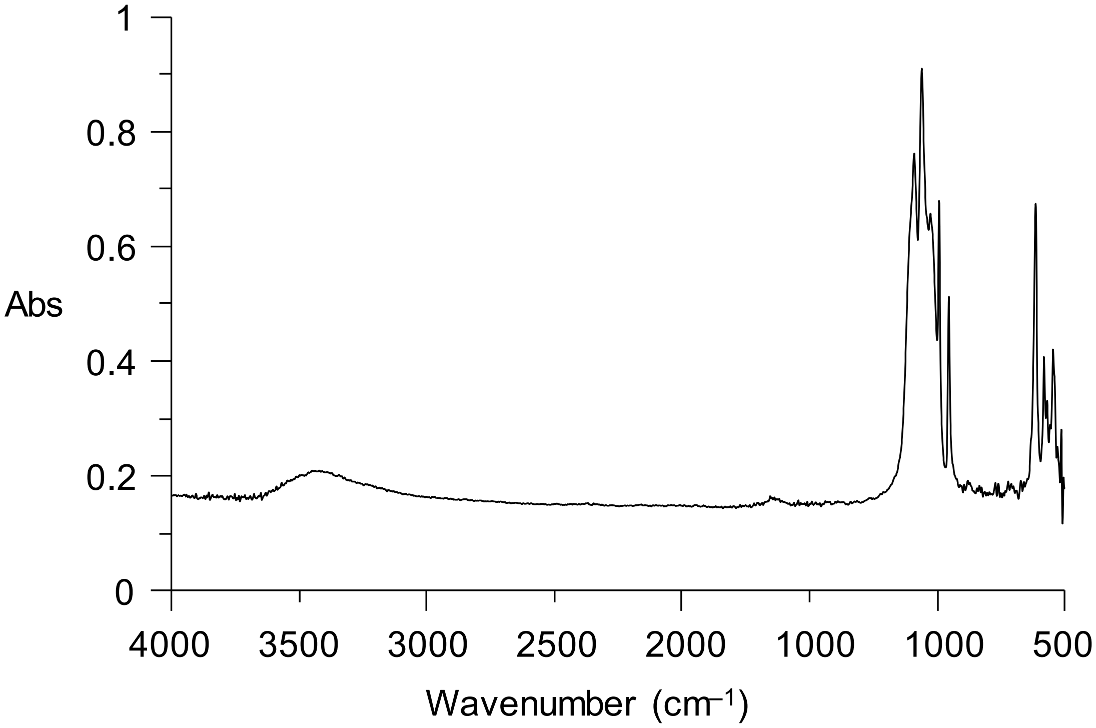

Fig. S1 IR spectrum of $CePO_4$.

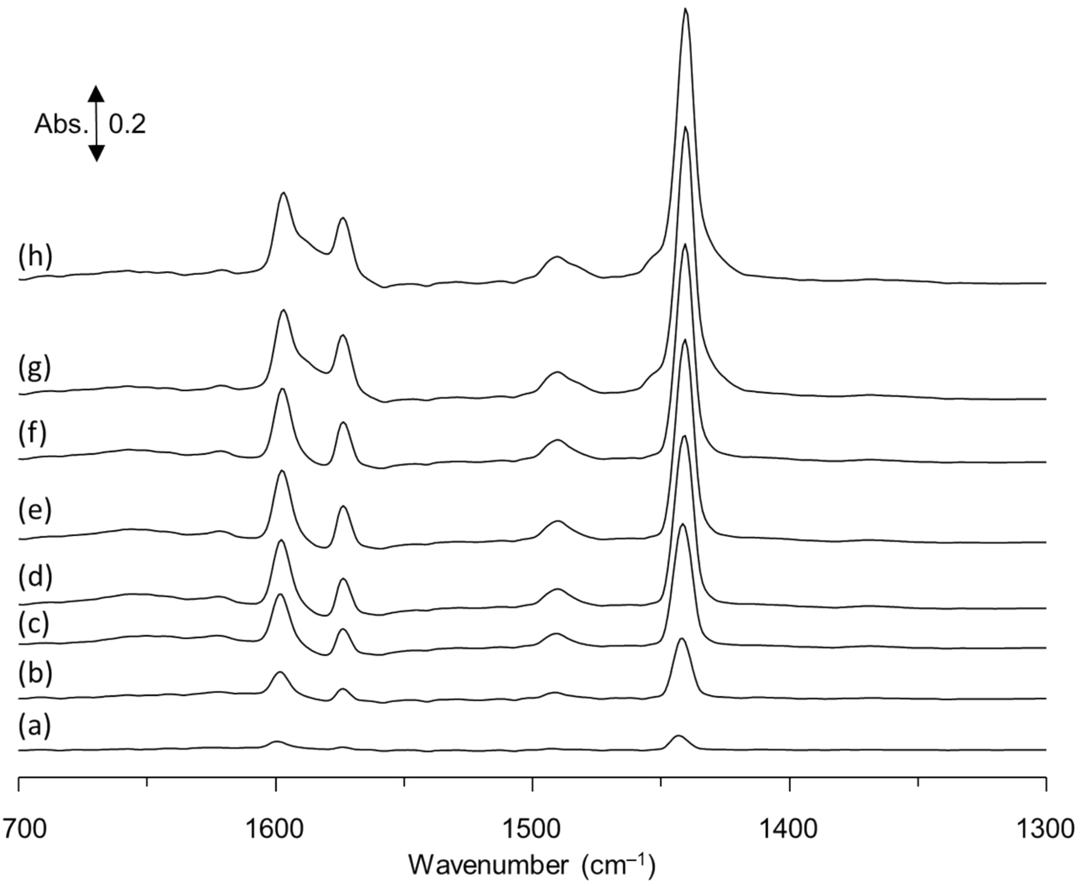

Fig. S2 Differential IR spectra for pyridine-adsorbed $CePO_4$. Gas-phase pyridine pressure: (a) 1.1×10^{-3}, (b) 1.6×10^{-3}, (c) 3.6×10^{-3}, (d) 1.0×10^{-2}, (e) 2.8×10^{-2}, (f) 5.2×10^{-2}, (g) 3.7×10^{-1}, and (h) 4.2×10^{-1} kPa.

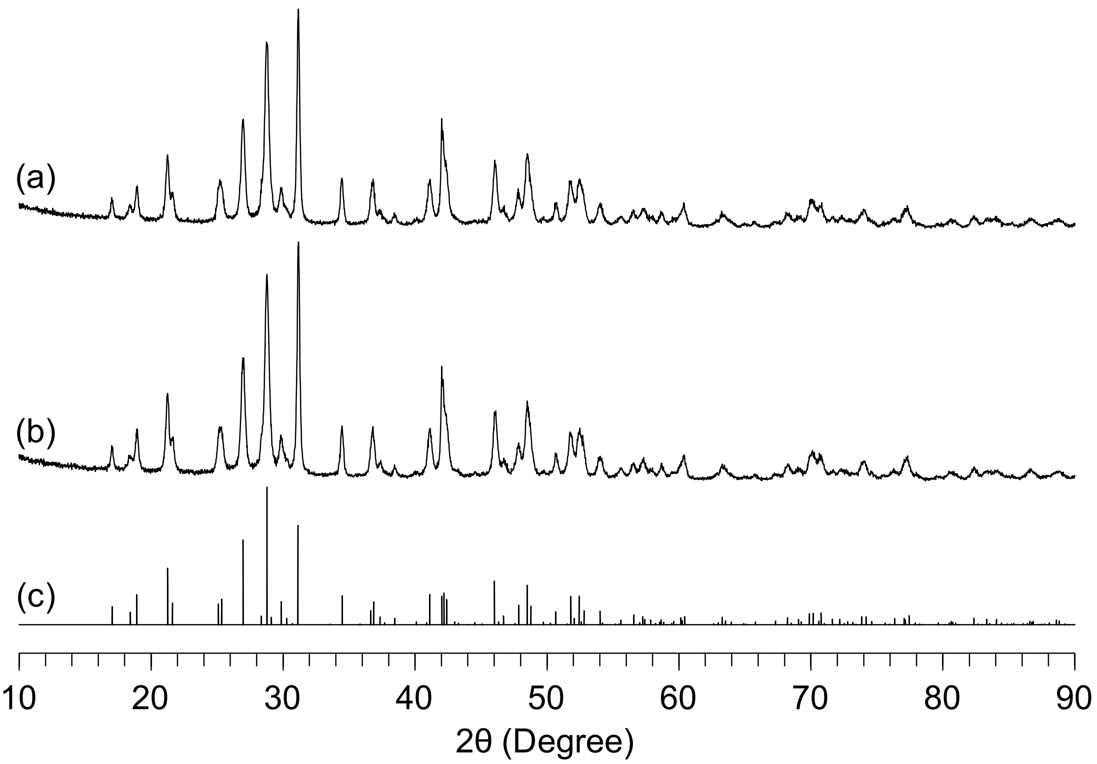

Fig. S3 XRD patterns for (a) $CePO_4$, (b) $CePO_4$ recovered after the reuse experiment, and (c) monoclinic $CePO_4$ (ICSD79748).

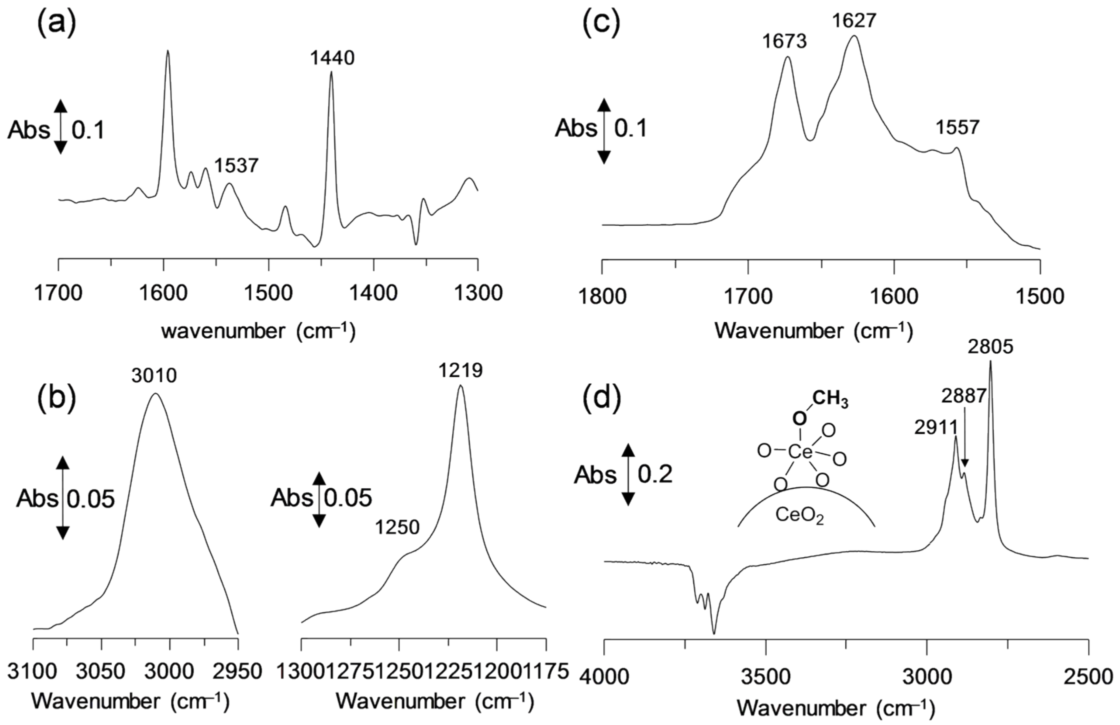

Fig. S4 Difference IR spectrum of (a) pyridine, (b) chloroform, (c) acetone, and (d) methanol-adsorbed CeO_2 at 25 ºC.
